# Alternative Geometries for 3D Bioprinting of Calcium Phosphate Cement as Bone Substitute

**DOI:** 10.3390/biomedicines10123242

**Published:** 2022-12-13

**Authors:** Jennifer Blankenburg, Johannes Vinke, Bianca Riedel, Sergej Zankovic, Hagen Schmal, Michael Seidenstuecker

**Affiliations:** 1 G.E.R.N. Center of Tissue Replacement, Regeneration & Neogenesis, Department of Orthopedics and Trauma Surgery, Medical Center, Faculty of Medicine, Albert-Ludwigs—University of Freiburg, Hugstetter Straße 55, 79106 Freiburg, Germany; 2 Institute for Applied Biomechanics, Offenburg University, Badstraße 24, 77652 Offenburg, Germany

**Keywords:** 3D printing, calcium phosphate cement, CPC, bone replacement, CDHA

## Abstract

In the literature, many studies have described the 3D printing of ceramic-based scaffolds (e.g., printing with calcium phosphate cement) in the form of linear structures with layer rotations of 90°, although no right angles can be found in the human body. Therefore, this work focuses on the adaptation of biological shapes, including a layer rotation of only 1°. Sample shapes were printed with calcium phosphate cement using a 3D Bioplotter from EnvisionTec. Both straight and wavy spokes were printed in a round structure with 12 layers. Depending on the strand diameter (200 and 250 µm needle inner diameter) and strand arrangement, maximum failure loads of 444.86 ± 169.39 N for samples without subsequent setting in PBS up to 1280.88 ± 538.66 N after setting in PBS could be achieved.

## 1. Introduction

The statistics of recent years on the number of implantations of artificial knee and hip joints in Germany and selected Organization for Economic Cooperation and Development (OECD) countries illustrate the necessity of biomaterial use in surgery. These implantations are some of the most commonly performed surgeries worldwide, mostly indicated by osteoarthritis, a degenerative form of arthritis [1]. In Germany, a trend toward an increased number of implantations of artificial hip and knee joints could be seen from 2005 to 2019, according to statistics from the Federal Statistical Office. A total of 243,477 implantations of artificial hip joints were performed throughout Germany in 2019 (the most reliable figures due to COVID-19). The number of artificial knee joints was 193,759 implantations for 2019 [2]. Similar high numbers can also be found for the EU [3].

The goal of bone regeneration is to restore lost bone material, both functionally and structurally. Current procedures often use autologous or allogeneic bone for regeneration [4]. A long segmental loss of substance can be bridged with microvascularly connected autogenous or allogeneic vascularized grafts. Segmental disruptions on long tubular bones can be treated with the help of distraction grafting [5]. In Germany, bone from the posterior and anterior iliac crests and parts of the fibula are used as the gold standard. However, this is only available in limited settings. For the patient, this increases the number of sites needed to heal, which results in more stress on the body [4]. In addition, the surgical time is prolonged because both the removal of the autogenous bone and the insertion into the defect are performed by means of one operation [6]. However, the use of bone bank materials has proven to be increasingly problematic in recent years due to adverse immune reactions and unwanted infections. Moreover, high costs significantly limit their use.

Infections mainly provide the impetus for the search for materials that adequately replace natural bone while being available in large quantities [5]. Biocompatible materials are an alternative. Depending on the nature of the defect, scaffolds are placed into the bone defect to provide a structural shape. Among various materials, calcium phosphate cement (CPC) is gaining much attention in medicine and dentistry due to its good biocompatibility, behavior as a bone substitute material, and ability to set on its own [7]. Calcium phosphate-based bone cements are produced from solid calcium phosphates mixed with a solution to achieve the precipitation of a calcium-deficient hydroxyapatite phase [8]. Calcium phosphate (CaP) is the most important biomaterial from the anthropocentric point of view. The inorganic component of physiological hard tissue in the human body is based on this class of substances. It is found as carbonaceous hydroxyapatite (HA) in bones, teeth, and tendons and gives them stability, hardness, and their function. Structurally, it is a low crystalline nonstoichiometric apatite phase. Human bone is composed of 50 to 60% biological apatite, which is a collective name for the minerals fluorapatite, chlorapatite, and HA [8]. Various formulations are known for CPC resulting in HA. As early as 1986, Brown and Chow developed a formulation consisting of dicalcium phosphate dihydrate (DCPD) (brushite), CaHPO_4_ 2H_2_O (dicalcium orthophosphate), CaHPO_4_ (DCP) mixed with tetracalcium phosphate (TTCP), and Ca(PO_4_)_2_O) using a molar ratio of 1:1 in an aqueous or dilute aqueous solution of phosphoric acid. The result was HA [9]. Using the same reactants but adjusting the molar ratio to 2:1, it was possible to prepare calcium-deficient HA (CDHA) [10]. The possibility of preparing CDHA from the hydrolysis of alpha-tricalcium phosphate (α-TCP) between 60° and 100° was described as early as 1976 by Monma and Kanazawa [11]. In the meantime, this formulation has been optimized so that lower temperatures can also be used for the formation of CDHA. During the setting reaction, α-TCP particles dissolve in the cement liquid, and CDHA crystals are formed by settling in the liquid [7]. Pure β-TCP, which is also very common as a biomaterial [12], is used in biomedicine in bone cements containing calcium phosphate, for example, as a mixture of β-TCP with HA as a bone substitute [13]. The α-TCP just mentioned is more reactive than β-TCP and can be hydrolyzed to a mixture of other CaP. In the medical field, it is used as CPC. In the present study, we used the CPC for the 3D printing of scaffolds, analogous to Vorndran et al. [14]. However, in this work we wanted to vary the geometric shapes and further adapt them to biological forms, i.e., without right angles, analogous to bone tissue (see Baino et al. [15]). Our hypothesis for the present work was that 3D-printed molded bodies that approximate biological shapes would be more mechanically stable and biocompatible because there are no 90° angles in biological systems (e.g., the skeleton). In addition, rotational symmetry should be present to print the layers with only 1° of rotation per layer so that the resulting scaffold is mechanically more stable compared to conventional scaffolds with 90° layer rotations.

## 2. Materials and Methods

### 2.1. Materials

Conical printer needles with 0.2 (article No.: BIO-008027) and 0.25 (article No.: BIO-008026) mm inner diameters were purchased from EnvisionTec (Gladbeck, Germany). The CPC paste for printing (20 mL, article No.: 087-020-PL) was purchased from Innotere (Radebeul, Germany). The DPBS (Gibco and Life Technologies as part of Thermo Fisher Scientific, Waltham, MA, USA) was purchased from ThermoFisher (article No.: 14190-094). Acetone ≥ 99.7% (article No.: CP40.4) was purchased from Carl Roth (Carl Roth GmbH + Co KG, Karlsruhe, Germany).

### 2.2. Three-Dimensional Printing

A 3D Bioplotter (EnvisionTec, Gladbeck, Germany) with a low-temperature printing head as well as conical needles made of polypropylene with inner diameters of 0.2 and 0.25 mm were used to print the different geometries. The CPC paste used was made by Innotere (Innotere, Radebeul, Germany). It consisted of synthetic calcium and phosphate salts finely dispersed in a biocompatible oil phase of short-chain triglycerides (caprycil/capric triglycerides) together with two further emulsifiers (polyoxyl-35-castor oil/cetyl phosphate). The triglycerides and the polyoxyl-35-castor oil (castor oil) were both based on pure vegetable raw materials.

#### 2.2.1. Optimizing Printing Parameters

To determine the optimum printing parameters, lines were first printed using the manual parameter tuning in Visual Machines software (EnvisionTec, Gladbeck, Germany). Pressure tuning for testing the pressure (bar) and speed tuning for testing the speed (mm/s) settings were available for this purpose. In both cases, several 5 cm long strands could be printed. A range of 0.6 to 2 bar was selected for the pressure. For the speed, a range of 8 to 23 mm/s was tested. A range of 0.25 to 0.45 mm was tested as the Z offset.

#### 2.2.2. Printing the Round Geometries

In previous works [16,17], cube-shaped geometries were printed using patterns from the Visual Machines software (EnvisionTec, Gladbeck, Germany). The goal was to print round structures and avoid 90° angles. First, straight spokes were designed as well as spokes with waves to avoid right angles altogether. To produce the round geometries with their own patterns, CAD models were created in several versions for inner needle diameters of 0.2 mm and 0.25 mm. For this purpose, a line model was designed as a 3D model using CREO 4.0 (PTC Inc., Boston, MA, USA). Spokes with straight lines and spokes with waves were created as inner patterns, all supported by smaller inner circles. These models were loaded into Perfactory RP software (EnvisionTec, Gladbeck, Germany) and stacked in 12 layers, each with one degree of rotation relative to the next layer. The scaffolds were set for 3 days at 37 °C in an incubator in a water-saturated atmosphere. After this time, half of the printed scaffolds were additionally incubated in Dulbecco’s Phosphate Buffered Saline (DPBS) for 1 week with daily changes of the DPBS.

### 2.3. Characterization of the Scaffolds

The characterization of the CPC scaffolds was performed by measuring the dimensions with a digital caliper (Burg-Wächter, Wetter-Volmarstein, Germany). Three-dimensional laser scanning microscopy (Keyence VK-X 200; Keyence, Osaka, Japan) was used to determine the surface roughness (center roughness) at 200- and 400-times magnification. XRD (Bruker D8 Advance, Bruker Corp., Billerica, MA, USA) and ESEM (FEI Quanta 250 FEG, FEI, Hilsboro, OR, USA) were also used. The mechanical strength and maximum failure load were determined using a Zwick Z005 universal testing machine (Zwick/Roell, Ulm, Germany). For this purpose, a compression test was performed with a preload of 1 N applied to the scaffolds, and the maximum failure load was determined at a traverse speed of 1 mm/s in a displacement-controlled manner.

### 2.4. Postprocessing of the Scaffolds

The postprocessing was the removal of the remaining oil from the scaffolds. For this purpose, the scaffolds were transferred individually into 15 mL reaction vessels (Falcon tubes), each containing 5 mL of acetone (Sigma Aldrich, St. Louis, MO, USA, article No.: 179124-1L), and the reaction vessels were then treated three times for 20 min in an ultrasonic bath. After each ultrasonic bath cycle, the scaffolds were transferred to new 15 mL reaction vessels with fresh acetone. The acetone was then evaporated under laminar flow over night. The next day, each scaffold was washed three times in double-distilled water and dried under laminar flow for 1 h.

### 2.5. Biocompatibility

For the cell culture experiments, the scaffolds were immersed for 3 × 20 min in acetone in an ultrasonic bath to remove the castor oil (part of the CPC paste from Innotere) to prevent the setting process before 3D printing. Afterwards, the scaffolds were incubated (in addition to incubation in PBS) in cell medium for at least one week with daily changes, since phosphoric acid is released as a by-product of the setting reaction, until a neutral pH value was reached. The scaffolds were then autoclaved.

MG-63 cells (ATCC, CRL 1427) were used for all biocompatibility tests. All tests were performed with 25,000 cells/100 µL per scaffold. Ten identical scaffolds per geometry variation were used per test, and all tests were repeated at least three times. The biocompatibility tests were carried out exclusively with the most mechanically stable scaffold types.

#### 2.5.1. Live/Dead Assay

On each scaffold, 100 µL of the medium was pipetted with 25,000 MG-63 cells/100 µL. The well plates were then incubated for 2 h at 37 °C and a CO_2_ saturation of 5% in an incubator. After two hours, 1 mL of the previously described specific cell medium was added to each well before incubating the well plates in the incubator for 3, 7, or 10 days. The staining solution was prepared by adding 2 mL of DPBS (article No. 14190-094, Gibco, Grand Island, NE, USA) to a Falcon tube (Greiner Bio-One International GmbH, Kremsmünster, Austria) and 4 µL of ethidium homodimer III (Eth D-III) solution (together with calcein part of the Live/Dead Cell Staining Kit II (PromoCell, Heidelberg, Germany)), according to the manufacturer’s protocol (PromoCell). A 1 µL amount of calcein dye was added after mixing the staining solution. All steps were performed in the dark to avoid the photobleaching of the staining solution and samples. To eliminate serum esterase activity, all samples at a time point had the medium removed, and the cells were washed. Staining was then performed according to a previously published protocol [18]. The evaluation was performed using an Olympus fluorescence microscope (BX51, Olympus, Osaka, Japan) at five different positions on the samples at 5× and 10× magnification.

#### 2.5.2. Cell Proliferation (WST-I)

Cells were again seeded on the scaffolds and as controls on Thermanox cover slips in the same number and concentration as in the previous biocompatibility tests. After two hours of incubation in the incubator at 37 °C and 5% CO_2_ saturation, the cells adhered to the scaffolds and Thermanox cover slips (as controls) so that the respective medium (1 mL) could be added. Plates were then incubated in the incubator for 3, 7, or 10 days. For this purpose, all medium was aspirated, and all wells were washed three times with PBS. Then, the scaffolds and Thermanox cover slips were transferred to a new 24-well plate. In the old well plate, 300 µL of DMEM medium without phenol red (additives: 1% FBP and 1% P/S) was added to each of the wells where the scaffolds and membranes had been. In the new well plate, 600 µL of the same medium was added to each of the wells containing the scaffolds and membranes. The blank contained only DMEM medium without phenol red (same additives) and was measured to account for background absorbance. Finally, 10% WST was added to all samples of the respective measurement time point (3, 7, or 10 d) and incubated for 2 h in the incubator. After 2 h, the absorbance was measured using a Spectrostar nano (BMGlabtech, Ortenberg, Germany) spectrometer at 450 nm.

#### 2.5.3. Cytotoxicity (LDH Assay)

LDH measurements were performed after 24, 48, and 72 h. In addition to the scaffolds, positive (Triton X, 100% toxicity) and negative controls (cells only, 0% toxicity) were used for the measurements at the different times. Both coated and uncoated scaffolds were also used. Cells were seeded onto the scaffolds and membranes in 100 µL of their medium (MG-63: 25,000 cells/100 µL). These were then incubated for 2 h in an incubator at 37 °C and 5% CO_2_ saturation. Following this, 1 mL of DMEM-F12 medium without phenol red was added to all wells with the additions of 1% P/S and 1% FBS. Since FBS in higher concentrations may induce background absorption, only 1% FBS was used. In the positive controls (C+), an additional 1% Triton X 100 was added to kill 100% of the cells. After 24 h of incubation in the incubator, 100 µL from each well were transferred to three new wells of a 96-well plate. Thus, from one well, three wells of 100 µL each were obtained. To ensure that the “blank” had the same concentration of phenol red, 100 µL of DMEM-F12 medium containing phenol red was added to this well prior to transfer. To evaluate the cytotoxicity, the Cytotoxicity Detection Kit solution was prepared. For this, 111.1 µL of catalyst solution was mixed with 5 mL of staining solution. Of this, 100 µL was pipetted into each well before incubating the well plate in darkness for 30 min. At the end of the 30 min, the absorbance at 490 nm could be measured using a spectrometer. The experiment was performed a total of four times.

### 2.6. Statistics

All data are presented as means ± standard deviations. Measured values were also analyzed using one-way analysis of variance (ANOVA) with a significance level of *p* < 0.05. Origin 2022 Professional SR1 (OriginLab, Northampton, MA, USA) was used for all statistical analyses.

## 3. Results

### 3.1. Three-Dimensional Printing Parameters

**Figure 1:**
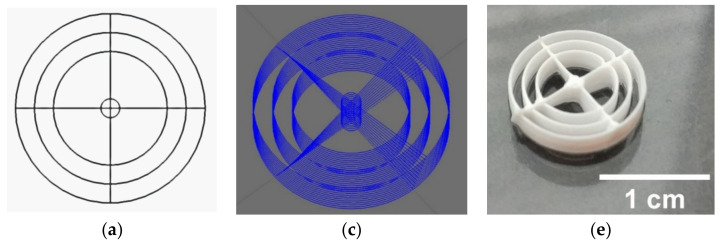

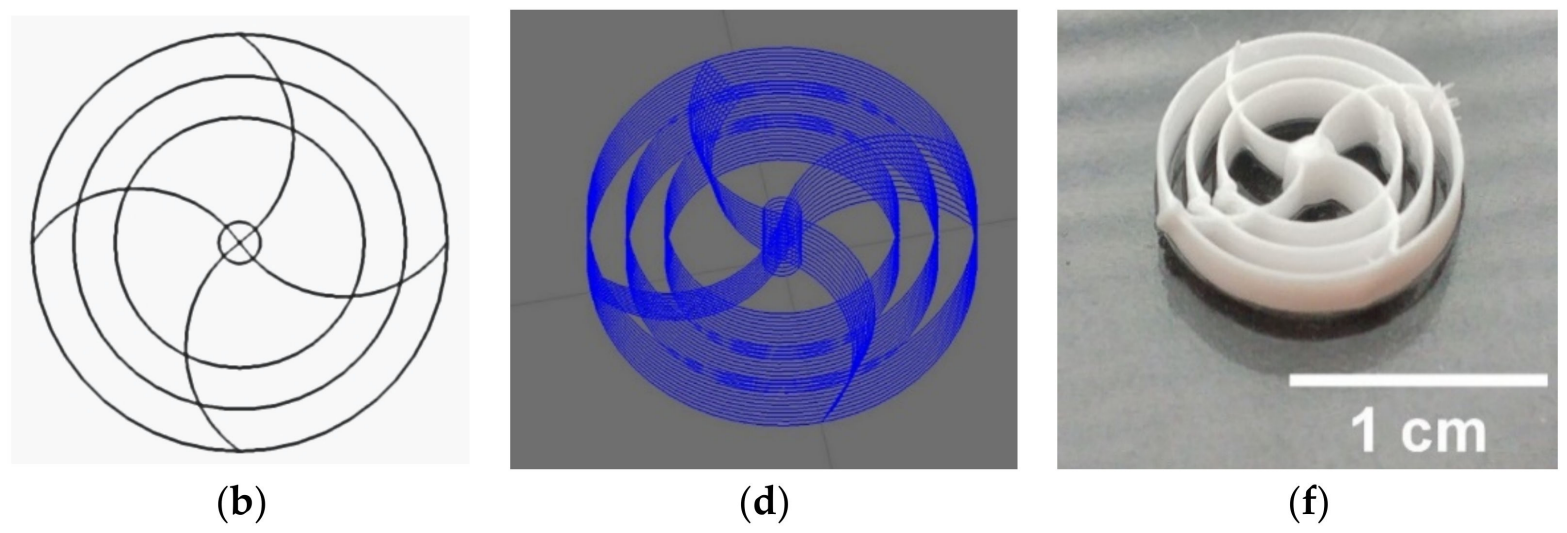
From concept to finished 3D print: (**a**,**b**) CAD models of the round structures with (**c**) straight spokes and (**d**) wavy spokes; stacked models with 12 layers and a rotation of 1° per layer for (**e**) straight spokes and (**f**) wavy spokes. CAD models created with CREO 4.0.

The z-offset of 0.3 mm showed the best results, both in pressure and speed tuning as well as in the final round geometries, for the 0.2 mm inner diameter needles. For the 0.25 mm inner diameter, this value shifted slightly to a z-offset of 0.25 mm. The results of the speed and pressure tuning showed significant differences for the lines compared to the final round geometries. The lines were best printed at a speed of 12 mm/s and 1 bar for the 0.2 mm needle and 9 mm/s and 1 bar for the 0.25 mm needle. This speed was too high for the geometries, so the speed was reduced to 3 mm/s while maintaining the same pressures. Figure 1 below shows the 3D models, stacked models with 12 layers each, and the final printed scaffolds with straight and wavy spokes. Table 1 summarizes the parameters used for all experiments.

### 3.2. Characterization of the Scaffolds

#### 3.2.1. Dimensions

**Figure 2:**
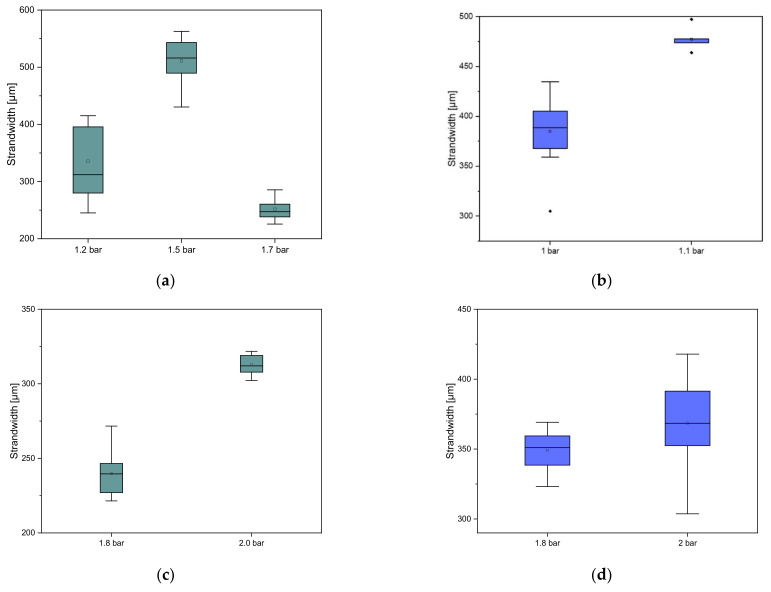
Effects of varying the pressure on the strand width of the scaffolds for (**a**) 250 µm straight spokes; (**b**) 250 µm wavy spokes; (**c**) 200 µm straight spokes; and (**d**) 200 µm wavy spokes. *n* = 10.

All samples with straight or wavy spokes printed with 200 µm or 250 µm needles were of the same size and on average 10.47 ± 0.10 mm in diameter. The printing speed and the pressure used were varied. The parameters are summarized in Table 1. The effects on the different scaffolds are summarized in Figure 2. For the strand thickness of the scaffolds, 250 µm straight spokes resulted in a strand thickness of 335.70 ± 61.58 µm at 6 mm/s and 1.2 bar. At 3 mm/s and 1.6 bar, a strand thickness of 257.23 ± 25.23 µm was observed, and at 3 mm/s and 1.5 bar a strand thickness of 547.95 ± 91.33 µm was observed. For the scaffolds with wavy spokes, the values at 6 mm/s and 1 bar were 384.95 ± 31.37 µm, and at 6 mm/s and 1.1 bar the values 477.23 ± 12.33 µm. When printing with 200 µm needles, the scaffolds with straight spokes at a printing speed of 3 mm and 1.8 bar gave a strand thickness of 248.43 ± 27.67 µm. In comparison, the value of the strand thickness at 3.5 mm/s was significantly higher, with a value of 314.51 ± 14.75 µm. For the scaffolds with wavy spokes, a value of 349.39 ± 13.98 µm was obtained for the strand thickness at 3 mm/s and 1.8 bar, while at 2 bar and 3 mm/s a value of 374.57 ± 33.50 µm was obtained.

For all further experiments, 3 mm/s and 1.7 bar were used for 3D printing with 250 µm needles for the scaffolds with straight spokes, while 6 mm/s and 1 bar were used for the scaffolds with wavy spokes. For the scaffolds produced with 200 µm needles, 3D printing was performed at 3 mm/s and 1.6 bar for the scaffolds with straight spokes and 3 mm/s and 1.8 bar for the wavy spokes.

#### 3.2.2. ESEM Measurements

**Figure 3:**
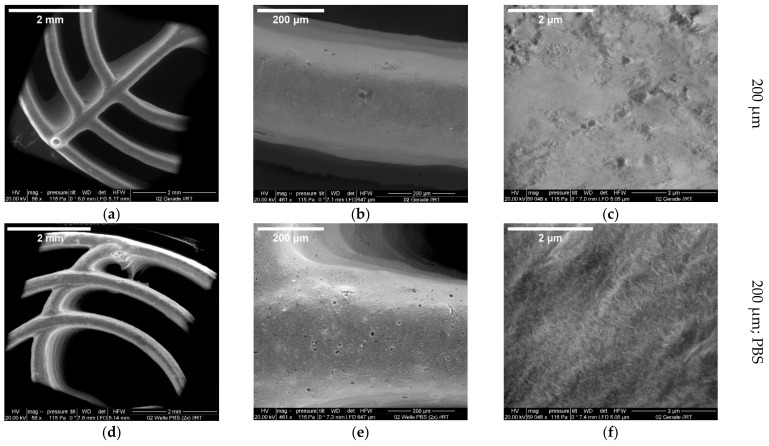
ESEM images of 3D-printed CPC scaffolds (200 µm) at increasing magnifications for an untreated straight spoke (**a**–**c**) and a wavy spoke after incubation for 1 week in PBS (**d**–**f**); images taken with an FEI Quanta FEG 250 ESEM with large field detector and 20 kV acceleration voltage at 115 Pa.

The magnified images show the structure of one of the outer rings. The roughness of the surface can already be seen at a scale of 30 µm. At a scale of 2 µm, the individual holes in the surface are recognizable, in which crystal-like structures can be seen. These crystal-like structures could be found in the scaffolds both with and without incubation in PBS or printed with the 200 µm or 250 µm nozzles (Figure 3).

**Figure A1:**
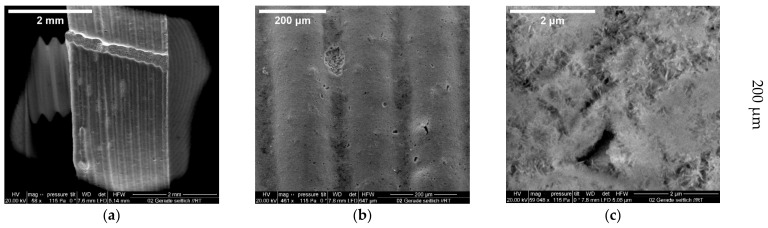
ESEM images of 200 µm straight spoke scaffold from the side at a fracture site. Magnified images (increasing magnification from (**a**–**c**)) show the scaffold from the side with the individual planes; accelerating voltage: 20 kV, vacuum: 114 Pa, large field detector.

In addition, broken scaffolds were also examined to determine the offset in the 1° layer rotation. The corresponding ESEM figures can be found in Figure A1.

#### 3.2.3. Three-Dimensional Laser Scanning Microscopy

**Figure 4:**
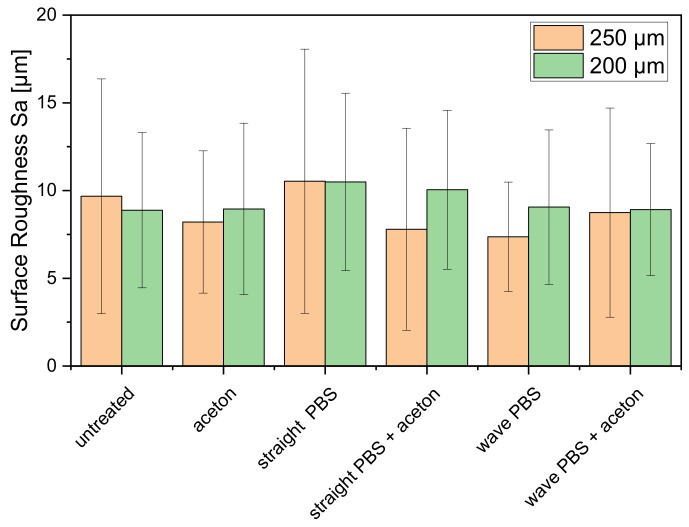
Surface roughness for the 3D-printed round geometries with straight and wavy spokes: untreated, washed in acetone, incubated in PBS for 1 week, and washed in acetone and incubated in PBS for 1 week, depending on the inner diameter of the needle used; *n* = 10.

For surface roughness, regardless of the conditions (untreated, washed in acetone, or stored in PBS) no significant difference could be found between the scaffolds regardless of the inner diameter of the needle. All measured values were in a similar range of 9.4 ± 1.2 µm (see Figure 4).

#### 3.2.4. XRD

**Figure A2:**
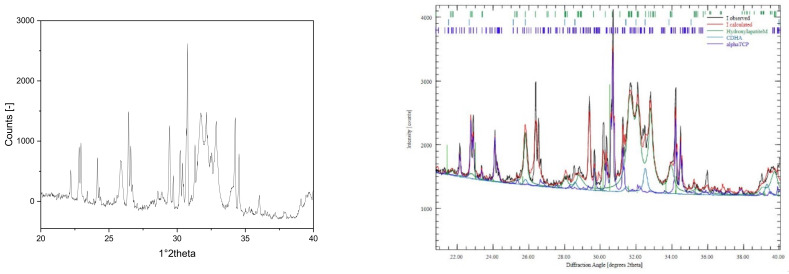
XRD pattern and Rietveld refinement calculation of the CPC paste; XRD pattern taken with Bruker D8 advance with 40 kV/40 mA, 1°2 theta/min, and step size of 0.02°2 theta; Rietveld refinement calculated with Profex 4.3.2.

The XRD spectra were analyzed using a Rietveld refinement analysis (Profex 4.3). There was no significant difference between the samples with or without incubation in PBS, while 90% HA, 7% α-TCP, and 3% CDHA could be detected (for further information, please see Figure A2 in Appendix A).

### 3.3. Biocompatibility

MG-63 cells (ATCC, CRL 1427) were used for all biocompatibility tests. All tests were performed with 25,000 cells/100 µL per scaffold. 10 identical scaffolds per geometry variation were used per test and all tests were repeated at least 3 times. 

#### 3.3.1. Live/Dead Assay

On each scaffold 100 µL of the medium were pipetted with 25,000 cells/100 µL of MG-63. The well plates were then incubated for 2 h at 37 °C and a CO_2_ saturation of 5% in an incubator. After two hours, 1 mL of the specific cell medium described previously was added to each well before incubating the well plates in the incubator for 3, 7 and 10 days. The staining solution was prepared by adding 2 mL DPBS (art. no. 14190-094, Gibco, Grand Island, NE, USA) to a Falcon tube (Greiner Bio-One International GmbH, Kremsmünster, Austria) and 4 µL ethidium homodimer III (Eth D-III) solution (together with calcein part of the Live/Dead Cell Staining Kit II (PromoCell, Heidelberg, Germany)) according to the manufacturer’s protocol (PromoCell). An amount of 1 µL of calcein dye was added after mixing the staining solution. All steps were performed in the dark to avoid photobleaching of staining solution and samples. To eliminate serum esterase activity, all samples at a time point had the medium removed and the cells washed. Evaluation was performed using an Olympus fluorescence microscope (BX51, Olympus, Osaka, Japan) at five different positions on the samples at 5× and 10× magnification.

#### 3.3.2. Cell Proliferation (WST-I)

Cells were again seeded on the scaffolds and as control on Thermanox cover slips in the same number and concentration as in the previous biocompatibility tests. After two hours of incubation in the incubator at 37 °C and 5% CO_2_ saturation, the cells adhered to the scaffolds and Thermanox cover slips (as control) so that the respective medium (1 mL) could be added. Plates were then incubated in the incubator for 3, 7 and 10 days. For this purpose, all medium was aspirated, and all wells were washed three times with PBS. Then, the scaffolds and Thermanox cover slips were transferred to a new 24-well plate. In the new well plate, 600 µL of the same medium was added to each of the wells containing the scaffolds and membranes. The blank contains only DMEM medium without phenol red (same additives) and was measured to account for background absorbance. Finally, 10% WST was added to all samples of the respective measurement time point (3, 7, 10 d) and incubated for 2 h in the incubator. After 2 h, the absorbance was measured using a spectrometer at 450 nm.

#### 3.3.3. Cytotoxicity (LDH Assay)

LDH measurements were performed after 24, 48 and 72 h. In addition to the scaffolds, positive control (Triton X, 100% toxicity) and negative controls (cells only, 0% toxicity) were used for the measurements at the different times. Both coated and uncoated scaffolds were also used. Cells were seeded onto the scaffolds and membranes in 100 µL of their medium (MG-63: 25,000 cells/100 µL). These were then incubated for 2 h in an incubator at 37 °C and 5% CO_2_ saturation. Following this, 1 mL of DMEM-F12 medium without phenol red was added to all wells with the additions of 1% P/S and 1% FBS. Since FBS in higher concentrations may induce background absorption, only 1% FBS was used. In the positive controls (C+), an additional 1% Triton X 100 was added to kill 100% of the cells. After 24 h incubation in the incubator, 100 µL from each well was transferred to three new wells of a 96-well plate. Thus, from one well, three wells of 100 µL each were obtained. To ensure that the “blank” had the same concentration of phenol red, 100 µL of DMEM-F12 medium containing phenol red was added to this well, prior to transfer. To evaluate the cytotoxicity, the Cytotoxity Detection Kit solution was prepared. For this, 111.1 µL of catalyst solution was mixed with 5 mL of staining solution. Of this, 100 µL was pipetted into each well before incubating the well plate in darkness for 30 min. At the end of the 30 min, the absorbance at 490 nm could be measured using a spectrometer. The experiment was performed a total of four times.

### 3.4. Mechanical Properties

**Figure 5:**
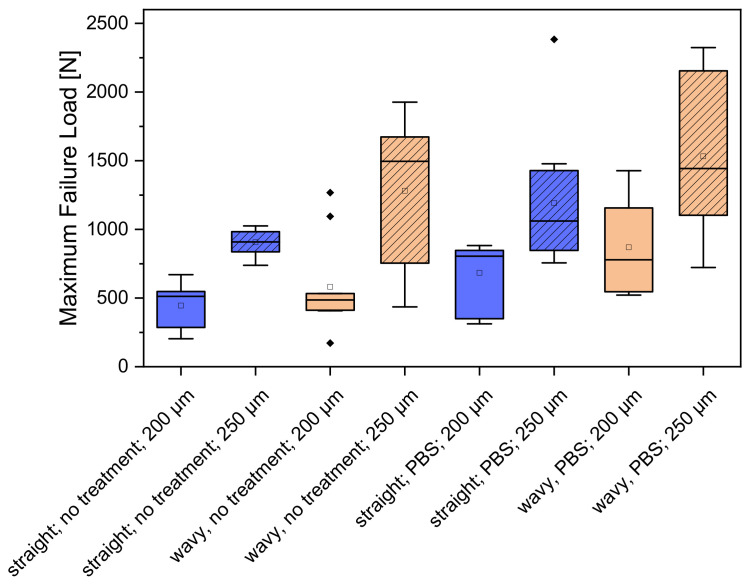
Overview of the maximum failure load for the different CPC scaffolds; *n* = 10.

**Figure 6:**
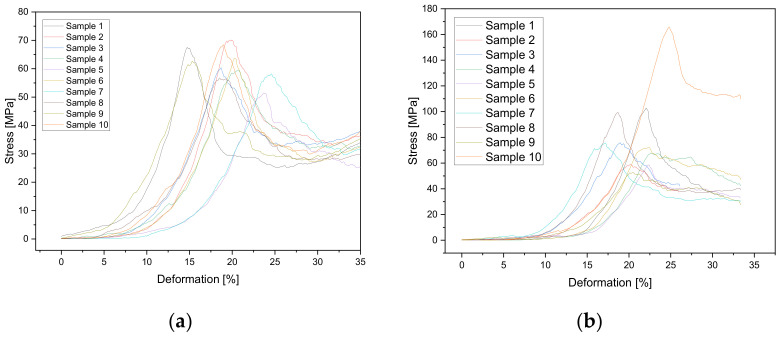
Stress–deformation curves for 3D-printed scaffolds with 250 µm strand width (*n* = 10) after incubation in (**a**) a saturated water atmosphere or (**b**) a saturated atmosphere and 1 week in PBS.

**Figure 7:**
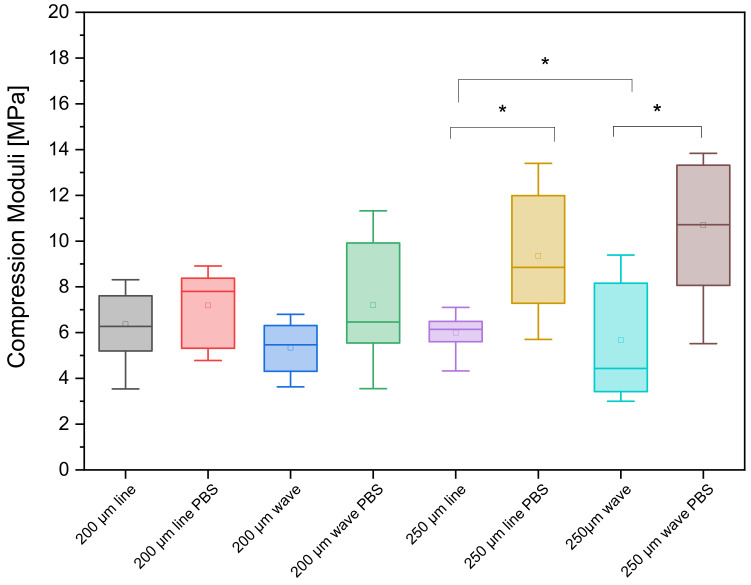
Comparison of the compression moduli of the different samples; * indicates statistical significance at *p* < 0.05.

**Figure A3:**
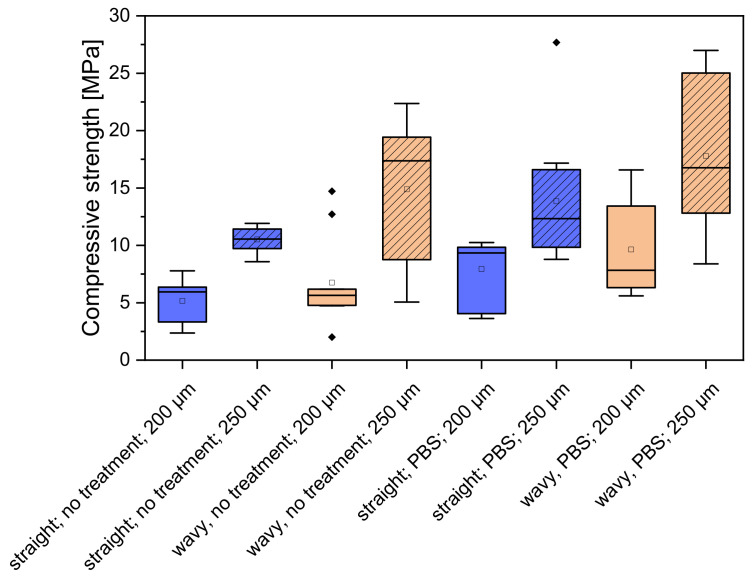
Compressive strength for the different scaffolds.

The scaffolds printed with a 250 µm needle showed a value up to 100% higher in relation to the maximum failure load of the scaffolds printed with a 200 µm needle. Incubation for 1 week in PBS also increased the maximum failure load by a factor of 2–4 compared to the untreated samples. Figure 5 shows the values of the maximum failure load for the different CPC scaffolds. For the straight spokes without PBS treatment, a maximum failure load of 444.86 ± 169.39 N was measured at the 200 µm needle inner diameter. The PBS-treated specimens reached a failure load of 1280.88 ± 538.66 N for 200 µm. Scaffolds printed with a 250 µm needle with straight spokes had slightly higher values of 905 ± 93 N when untreated and 1531.53 ± 611.48 N after incubation in PBS. A complete overview of the results is shown in Figure 5. Comparing the scaffold types (straight and wavy spokes), there was no significant difference between the two untreated samples for the scaffolds printed with a 200 µm strand width. For the samples incubated in PBS, there was a significant difference. For the samples with a 250 µm strand width, there was a significant difference between the untreated scaffold types (straight and wavy spokes) and the treated samples. The comparison between untreated and PBS-incubated samples also showed significant differences for both straight and wavy spoke scaffolds. In the curves of the deformation, as a function of the standard force, it was noticeable that the sample with the wavy spokes covered larger deformation ranges. The scaffolds produced using the 0.2 mm needle with straight spokes broke at a deformation between 15 and 25%, while the scaffolds with wavy spokes failed between 15 and 40% deformation when untreated and after DPBS treatment, respectively. For the 0.25 mm needle, the scaffolds with the straight spokes broke between 15 and 25% deformation and the scaffolds with wavy spokes broke at deformations between 35 and 50% (please see Figure 6 and Table 2). The calculated values of the compression moduli varied between 5.67 ± 2.46 and 10.7 ± 3.2 MPa. It was found that the influence of the PBS treatment did not have a strong effect on the compression moduli of the scaffolds with straight spokes and 200 µm strand widths. Significant differences (*p* < 0.05), however, existed for the scaffolds produced with 250 µm strand widths (cf. Table 2 and Figure 7 and Figure A3). For simplicity, the straight spoke is referred to as “line” and the wavy spoke is referred to as “wave” for the following tables and figures; +PBS indicates incubation in PBS for 1 week.

#### 3.4.1. Live/Dead Staining

**Figure 8:**
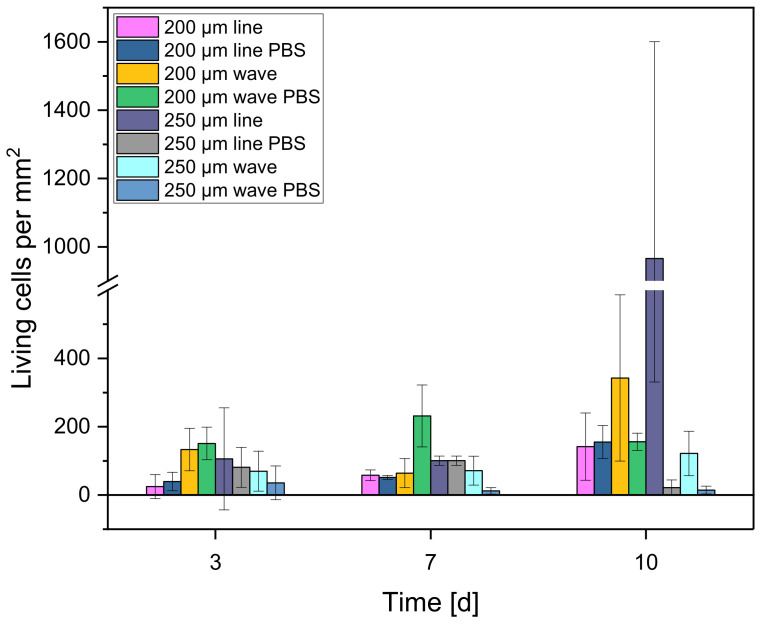
Number of living cells/mm^2^ on the different scaffolds after 3, 7, and 10 days.

**Figure 9:**
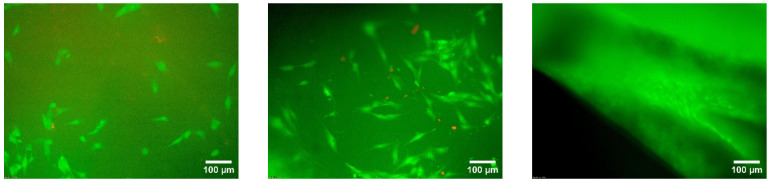
Fluorescence microscopic images of the surface of the 200 µm wave PBS sample with 10× magnification, showing dead cells (red) and living cells (green); from left to right: days 3, 7, and 10. Images taken with an Olympus BX-53 fluorescence microscope.

The number of living cells/mm^2^ showed different patterns. The 200 µm line and 250 µm wave scaffolds were the only two samples where the number of living cells increased continuously from day 3 to day 10: 24.89 ± 35.03 cells/mm^2^ on day 3, 57.91 ± 15.60 cells/mm^2^ on day 7, and 141.8 ± 98.58 cells/mm^2^ on day 10 (200 µm line) and 69.82 ± 58.54 cells/mm^2^ on day 3, 71.44 ± 42.22 cells/mm^2^ on day 7, and 121.78 ± 64.89 cells/mm^2^ on day 10 (250 µm wave) (cf. Figure 8 and Figure 9).

#### 3.4.2. Cell Proliferation (WST-I)

**Figure 10:**
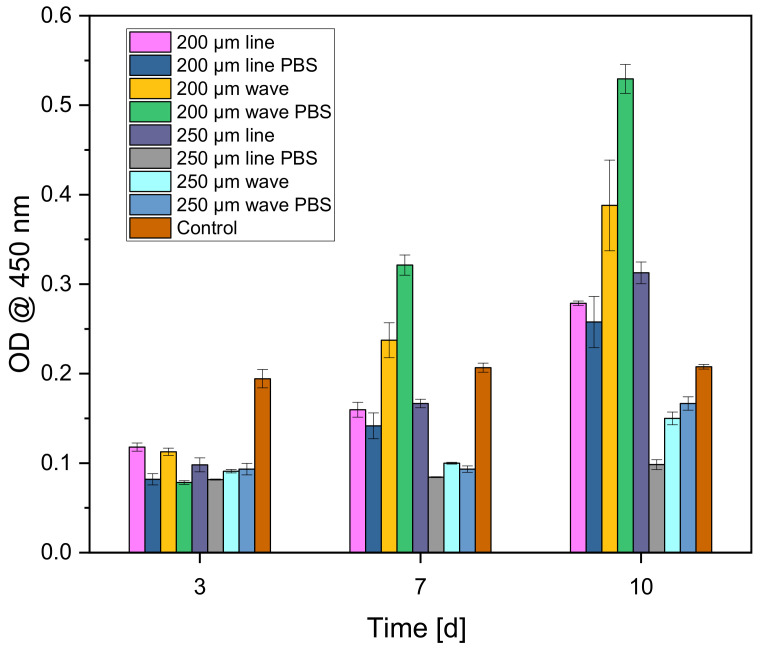
Cell proliferation for the different CPC scaffolds over 10 days. A β-TCP ceramic from Curasan served as a control.

Figure 10 shows that the cells proliferated on the different scaffolds. The scaffolds previously incubated in PBS showed the highest increases. The control (3D cell culture on β-TCP ceramics from Curasan) showed a very slow proliferation rate.

#### 3.4.3. Cytotoxicity (LDH)

**Figure 11:**
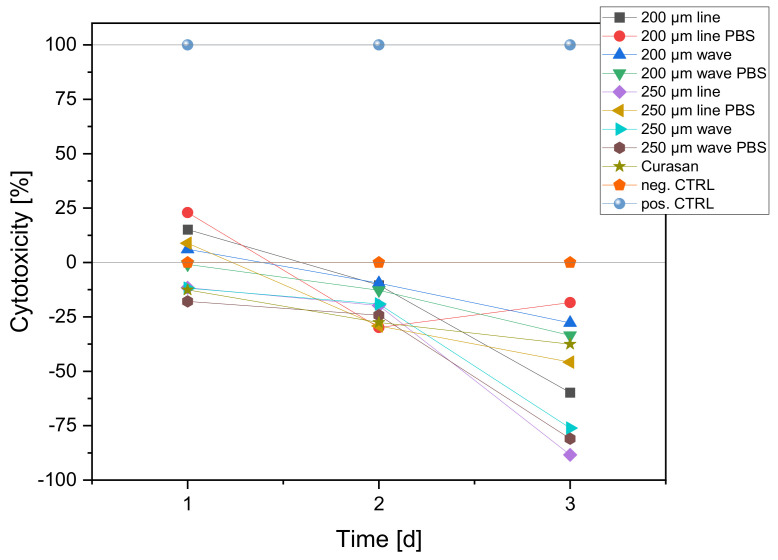
Cytotoxicity of the different scaffolds after 72 h. The positive control (pos. CTRL) was a 1% Triton X solution on cells, and the negative control (neg. CTRL) was cells. Curasan was the 3D cell culture control.

The cytotoxicity of the samples began with some scaffolds with values up to 25% after 24 h but then dropped to 0% for all samples (all values less than 0% correspond to 0% cytotoxicity) from 48 h to 72 h. The positive control (pos. CTRL) was a 1% Triton X solution on cells, and the negative control (neg. CTRL) was cells. Figure 11 shows the cytotoxicity for all scaffolds.

## 4. Discussion

### 4.1. Manual Parameter Tuning

The aim of the parameter tuning was to find the appropriate parameters for printing the geometries with the needle inner diameters of 0.2 mm and 0.25 mm. Good results were obtained with the aid of the 3D laser scanning microscope. For an inner needle diameter of 0.25 mm, lines similar to the inner needle diameter (215.03 to 252.04 µm) could be printed with a Z-offset of 0.3 to 0.35 mm, a speed of 10 to 12 mm/s, and a pressure of 0.6 to 1.1 bar. Even for the needle inner diameter of 0.2 mm, lines could be printed that were similar in strand thickness to the inner diameter (185.71 to 213.64 µm). Thus, with an offset of 0.3 mm, a speed of 8 to 10 mm/s, and a pressure of 0.8 bar to 1.1 bar, good results could be achieved. Here, the assumption can be made that increasing pressure results in thicker lines, which can be corrected with a high speed, resulting in thinner lines. In a study by Luo et al. [19], his correlation with different needle diameters became clear. When checking the strand thicknesses before and after setting, it was found that the thicknesses of the lines increased after setting in the incubator, with individual exceptions. Thus, the strands changed between values of 80.29 µm and 2.79 µm. Here, it seems that there was a slight swelling of the samples or that there was an influence of gravity so that the strands sank and became thicker. However, errors may have occurred if measurements were not made at exactly the same points on the scaffolds. There were also exceptions where the strands were thinner after setting. In studies by Luo et al. [19] and Lode et al. [20], almost no or no change in size was found during the manufacturing process.

### 4.2. Printing the Scaffolds

In other studies [20], CPC scaffolds were printed with inner needle diameters of 610 µm and 838 µm. Similar to our study, the paste came from Innotere GmbH. At a pressure of 2.2 bar and a speed of 700 mm/min (11.67 mm/s), scaffolds with up to 34 layers could be printed with a strand thickness of 400 µm with an inner needle diameter of 838 µm. Here, a significantly higher speed and a higher pressure were used. Nevertheless, strand thicknesses lower than the inner needle diameter could be achieved. In a study by Abarrategi et al. [21], an inner needle diameter of 250 µm was also used to print HA and β-TCP scaffolds. They achieved a strand thickness of only 210 ± 10. In this case, it was also possible to achieve a strand thickness smaller than the inner needle diameter. In another study by Richter et al. [22], CPC scaffolds with a size of 12 × 12 mm were printed with an inner needle diameter of 410 µm. Here, the CPC paste also came from the company Innotere GmbH. At a pressure of 1.2 bar and a speed of 5.5 mm/s, a layer thickness of 0.3 mm was achieved. Similar pressures and speeds were used as in this work. It was thus achieved that the strand thickness was lower than the internal needle diameter. Wu et al. [23] also printed CPC with layers of 100 µm. Unfortunately, they did not specify exactly what kind of needle was used for printing. Li et al. [24] printed cylindrical scaffolds as we did, but unfortunately, except for the printer model, no information about the parameters of the printing process was given. Ahlfeld et al. [25] used needles with 250–410 µm inner diameters and printing speeds between 4 and 12 mm/s with pressures between 2.5 and 4.5 bar. However, they did not investigate pure CPC scaffolds but biphasic scaffolds of CPC and alginate. Nevertheless, they also printed round scaffolds analogous to the present work.

### 4.3. Mechanical Properties

In the mechanical compression tests, a difference was found between the untreated scaffolds and the scaffolds after 7 days in DPBS. However, the results showed significance of the mean values only for the 200 µm straight spoke and 200 µm straight spoke PBS geometries. For the other constructs shown here, no significance was observed. Storing the scaffolds in an aqueous solution for a longer time probably continued the setting reaction and created more bonds as well as the formation of more HA crystals. In the case of the deformations as a function of the acting standard force for the scaffolds with the needle inner diameter of 0.2 mm, it was noticeable that groups were formed in the graphs. The scaffolds showed similar deformations, but the forces required for this differed so that two groups of deformations were formed in most cases. It is possible that the tube with CPC paste was changed here when printing these scaffolds, which could have resulted in a slightly different composition of ingredients. Even minor changes could have an influence on the properties of the scaffolds. In other studies, such as Richter et al. [22], it was found that the linear elastic deformation of the CPC scaffolds ended with a fracture, followed by an increasing compressive stress, an increasing load, and a renewed fracture. Values of up to 5.5 ± 1.4 MPa were achieved. Li et al. [24] used cylindrical scaffolds as bone substitutes analogous to the present work. However, they did not perform any mechanical characterization. In another study by Lode et al. [20] an ultimate strength of 6.1 ± 1.8 MPa was obtained. However, it was also noted that the mechanical properties of porous scaffolds are highly dependent on the pore size, morphology, distribution, and orientation. In a previous study [26], β-TCP scaffolds were inversely printed with different pore sizes. Depending on the pore size (in µm), the scaffolds broke at 543.6 ± 35 N (500 µm), 243.4 ± 34.1 N (700 µm), and 117.5 ± 43.4 N (1000 µm). Compared to the inversely printed scaffolds, we reached similar values, with maximum failure loads between 500 and 1400 N. Muallah et al. [17] achieved compressive strengths of 5.2 ± 0.6 to 31.3 ± 6.8 MPa for square scaffolds, depending on the strand spacing and pore sizes. Ahlfeld et al. [25] incubated their biphasic scaffolds in CaCl_2_ and achieved an increase in compressive strength from 0.5 to 0.7 MPa compared to the untreated specimens. Compared to this, the compressive strengths of our scaffolds were in a similar range to Mullah et al. [17] between 5 and 25 MPa. In our other works [27], we also investigated the compressive strength of PCL scaffolds with different inner and outer geometries. They reached 91.4 ± 1.4 MPa and were even smaller dimensioned than the CPC scaffolds. However, we had not yet used a layer rotation of 1° in that work.

### 4.4. ESEM and XRD

The scanning electron microscope images were able to clarify how the surface of the scaffolds behaves. The crystals of HA, which form during the setting reaction, were clearly visible. An XRD analysis was used to prove that these crystals were HA. In addition, a difference between the untreated scaffolds and those after 7 days in DPBS was observed in these samples. Thus, the DPBS apparently caused the setting reaction to continue, and more HA was formed, which was evident from the greater number of pits with crystals and less smooth surfaces. In addition, a clear difference was found in the areas where the planes stacked like stairs. Here, the surface was covered with crystals similar to a lawn. This type of turf was not found on the lateral surfaces. In a study by Richter et al. [22], the same crystalline structures were found on the CPC scaffolds when images were taken with the ESEM. Here, too, the formation of CDHA was assumed. Li et al. [24] printed and also examined their CPC scaffolds, but unfortunately no information was given on the composition or the dimensions of the individual strands other than an EDX analysis for the distribution of silicon (Si^+^) ions. Ahlfeld et al. [25] performed an ESEM/EDX analysis to investigate microcracks in the scaffolds caused by the incubation of “green bodies” in a CaCl_2_ solution. Such microcracks did not occur during our solidification (first in a water-saturated atmosphere followed by incubation in DPBS).

### 4.5. Biocompatibility

An optimal pH is recommended for cellular processes. This physiological value is 7.4. This must be kept within narrow limits of 7.2 to 7.6 by means of sufficient control mechanisms and adequate buffering [28]. With the values measured here, this optimum could be maintained. In a study by Klammert et al. [29], a physiological pH value could always be measured when TCP scaffolds forming brushite and, in further processing, monetite were measured. The pH developed over a period of 12 days but was at no time below 7.2 and was significantly lower than that of the cell medium.

The live/dead evaluation also showed that cell growth was possible on the scaffolds. For most scaffolds, most attachment and growth of cells was found on the middle part of the scaffold, where the spokes meet. In a study by Moreau et al. [30], a live/dead evaluation was performed after one day with calcein-AM and ethidium-homodimer-I. Few dead cells were found, and the number of live cells was 173 ± 42 cells/mm^2^. Overall, 96% of the found cells were alive. In this work, the number of live cells found on the scaffolds was 79.73 ± 26.28%.

Using the WST assay, it was demonstrated that cell growth had occurred on the scaffolds. It is possible that the surface roughness of the scaffolds is not sufficient for the cells to adhere to them. In addition, the amount of cell medium could also be reduced to prevent excessive medium from inhibiting cells from spreading in the cell culture plate.

A similar picture was found in our previous study with inverse β-tricalcium phosphate scaffolds [31]. According to this study, the web width of the constructs with smaller pore sizes had a direct effect on the proliferation rate. Similar observations were also made by Fink et al. [32]. They observed preferential occupancy with the increasing area of the adhesion site.

LDH activity measurements also showed that the scaffolds did not appear to be toxic to the cells. All scaffolds showed a cell cytotoxicity of 0% on days 7 and 10 of the evaluation. This may have been due to the contamination of the sample, as it showed a cell cytotoxicity of 0% on the other days of the evaluation. In a study by Lode et al. [20], the cell medium was incubated with CPC scaffolds for 24 h. This medium was then added to cultured cells and LDH evaluations were performed on days 1 and 7. Again, after a reduction in cells on day 1, a proliferation of cells was detected on day 7. As in this work, printed CPC scaffolds were also incubated in medium for 24 h and then covered with cells. LDH activity measurements were performed here on days 1, 6, 12, and 19. Initially, there was a lag phase, even a decrease between days 1 and 6. However, this also showed an increase in cells.

## 5. Conclusions

With regard to the objective of using alternative geometries for the 3D bioprinting of CPC scaffolds, strand diameters with two different needle inner diameters of 0.2 and 0.25 mm could be printed. Constructs with straight and corrugated spokes with 12 layers with 1° of rotation each could be produced. During the tests to evaluate the mechanical properties of the scaffolds, it was found that incubation in DPBS resulted in an increase in the maximum load and a significant increase in the compressive strength. Thus, the alternative geometries of CPC for 3D printing could also be interesting for other 3D printing applications of scaffolds for bone replacement where mechanically stable constructs are needed. In addition, the printed CPC scaffolds proved to be biocompatible.

## Figures and Tables

**Table 1:** Printing parameters for the different scaffolds.

Sample	Offset	Printing Speed	Pressure	Preflow	Postflow
250 µm straight *	240 µm	3 mm/s	1.2 bar	0.3 s	0.0 s
250 µm straight	240 µm	3.5 mm/s	1.2 bar	0.3 s	0.0 s
250 µm straight	240 µm	6 mm/s	1.2 bar	0.3 s	0.0 s
250 µm wave *	240 µm	3 mm/s	1 bar	0.3 s	0.0 s
250 µm wave	240 µm	6 mm/s	1 bar	0.3 s	0.0 s
200 µm straight *	180 µm	3 mm/s	1.6 bar	0.3 s	0.0 s
200 µm straight	180 µm	3.5 mm/s	1.6 bar	0.3 s	0.0 s
200 µm wave *	180 µm	3 mm/s	1.8 bar	0.3 s	0.0 s
200 µm wave	180 µm	3 mm/s	2 bar	0.3 s	0.0 s

The final setup, where the strand width = the inner diameter of the needle used, is marked with *.

**Table 2:** Summary of the compression moduli for the different scaffolds (*n* = 10).

Compression Moduli (MPa)							
200 µm		200 µm + PBS		250 µm		250 µm + PBS	
Line	Wave	Line	Wave	Line	Wave	Line	Wave
6.36 ± 1.58	7.19 ± 1.59	5.34 ± 1.14	7.19 ± 2.57	5.99 ± 0.78	5.67 ± 2.46	9.35 ± 2.72	10.7 ± 3.2

## Data Availability

The data presented in this study are available on request from the corresponding author.
